# Validation of cadaver-based trauma surgery training for lifelong skill development

**DOI:** 10.1186/s13017-025-00608-4

**Published:** 2025-05-29

**Authors:** Soichi Murakami, Toshiaki Shichinohe, Yo Kurashima, Kazufumi Okada, Yusuke Tsunetoshi, Ryoji Iizuka, Wataru Ishii, Kenji Kandori, Shinichiro Irabu, Naoki Shinyama, Hiroshi Homma, Masahiko Watanabe, Satoshi Hirano

**Affiliations:** 1 Center for Education Research and Innovation of Advanced Medical Technology, Department of Gastroenterological Surgery II, Hokkaido University Hospital, Sapporo, Japan; 2 Cadaver Trauma Surgery Training and Research Group, Tokyo, Japan; 3 Department of Gastroenterological Surgery II, Hokkaido University, Rm. 112-3, 7F Medical and Dental Research Building, N13 W7, Kita-Ku, Sapporo, Hokkaido 060-8648 Japan; 4 Clinical Simulation Center, Hokkaido University Graduate School of Medicine, Sapporo, Japan; 5 Data Science Center, Promotion Unit, Institute of Health Science Innovation for Medical Care, Hokkaido University Hospital, Sapporo, Japan; 6 Department of Surgery, Teine Keijinkai Hospital, Sapporo, Japan; 7 Emergency and Critical Care Center, Kyoto Daini Red Cross Hospital, Kyoto, Japan; 8 Department of Acute Care Surgery, Seirei Hamamatsu General Hospital, Hamamatsu, Japan; 9 Department of Acute Care Surgery, Sakai City Medical Center, Sakai, Japan; 10 Department of Emergency and Critical Care Medicine, Tokyo Medical University, Tokyo, Japan; 11 Department of Anatomy, Faculty of Medicine, Hokkaido University Graduate School of Medicine, Sapporo, Japan

**Keywords:** Cadaver-based educational seminar for trauma surgery, Lifelong cadaver, Surgical training, Self-assessment of confidence levels

## Abstract

**Background:**

The decline in trauma cases and the increase in non-surgical treatments have reduced opportunities for trauma surgery training. This study examined the effectiveness of Cadaver-Based Educational Seminar for Trauma Surgery (C-BEST) as a lifelong educational tool for novice and experienced clinicians.

**Methods:**

From 2017 to 2023, 117 clinicians with varying levels of experience participated in the C-BEST program at Hokkaido University. Participants included novice clinicians (median years post-graduation: 5) and experienced clinicians (median years post-graduation: 19). Each participant assessed their confidence in 21 trauma techniques before, immediately after, and 6 months post-course using a self-assessment of confidence levels (SACL) scale.

**Results:**

The analysis showed significant improvement in SACL scores immediately after the course, with confidence levels remaining sustained 6 months later. Novice clinicians demonstrated substantial skill acquisition, whereas experienced clinicians reported the reinforcement and refinement of existing skills.

**Conclusions:**

C-BEST seems valuable as a training tool for the acquisition and retention of trauma surgery skills, addressing practical needs in trauma care. C-BEST provides an effective and sustained approach to trauma surgery skill development and retention across career stages. Further research on its long-term impact and applicability in diverse clinical settings is recommended.

## Background

Trauma surgery is a fundamental skill for all surgeons. However, owing to a decline in traffic accidents and advances in non-surgical treatments, such as interventional radiology, training opportunities in clinical settings are diminishing [1–3]. Nevertheless, trauma cases have not entirely disappeared, and surgeries are still performed every year [4]. In urban areas, hospitals with specialized trauma surgeons can maintain high standards of trauma care through centralized services [5, 6]. However, in rural areas, delivering timely treatment and surgical interventions is challenging owing to transportation delays. Moreover, general surgeons infrequently handle severe trauma cases, sometimes only a few cases annually [7].

Recently, the utility of cadaver-based surgical training has gained recognition, and various medical fields have adopted this training [8–11]. In trauma surgery, training using cadavers is considered valuable for beginners seeking to acquire trauma surgical skills [8, 10, 12–16] and has become essential as opportunities to acquire these skills in real-life settings have dwindled. Among training programs, our program, Cadaver-Based Educational Seminar for Trauma Surgery (C-BEST), was primarily designed for novice surgeons and emergency physicians, and its effectiveness has been validated [12–15]. However, many experienced surgeons and emergency physicians also participate in C-BEST to hone their skills, with instructors often joining for this purpose. In this study, we analysed whether C-BEST is an effective means of lifelong education.

## Methods

### C-BEST settings

**Fig. 1:**
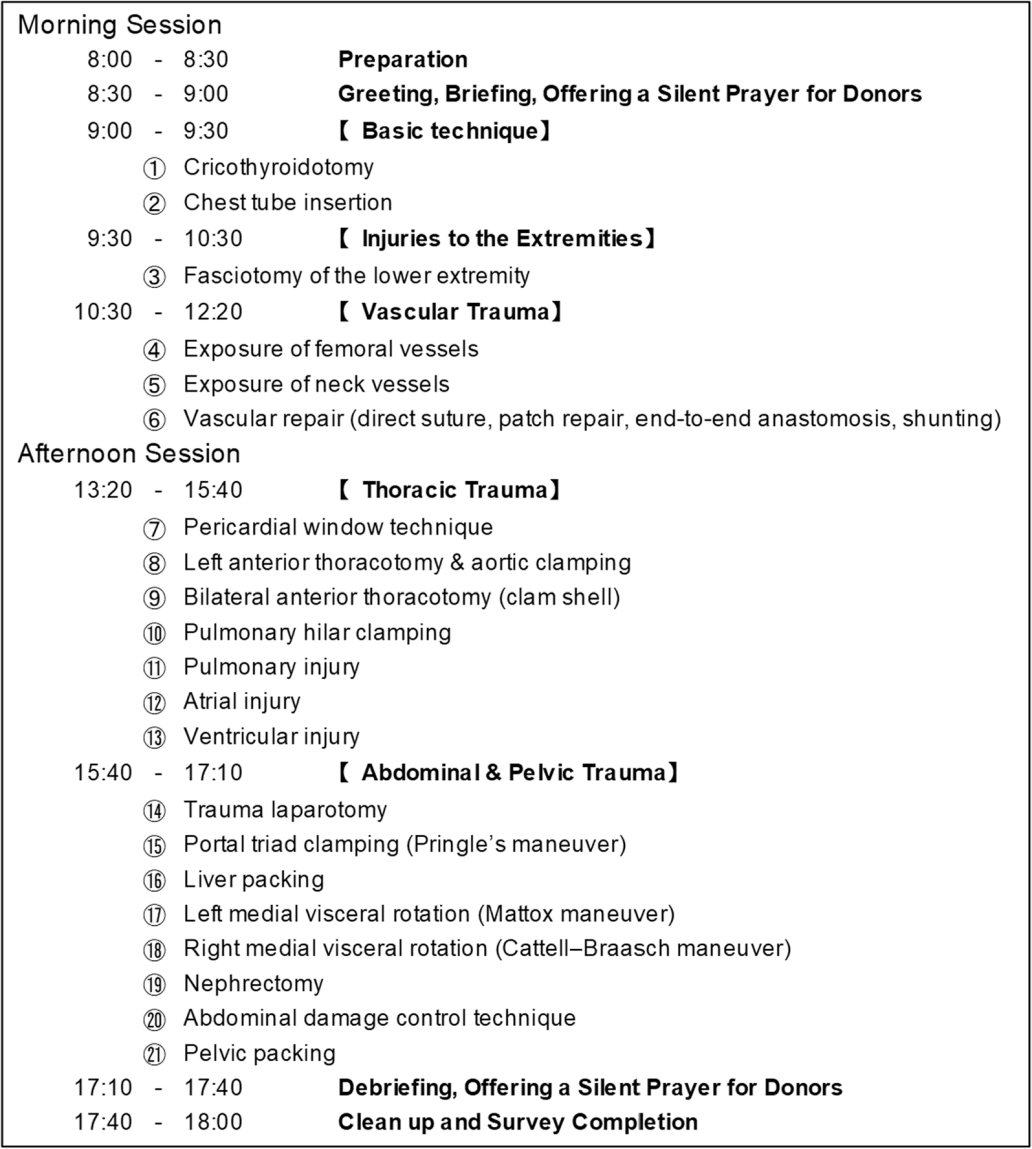
Timeline of the C-BEST program showing the detailed schedule of the eleven 1-day seminars and the trauma surgery techniques taught during each session

C-BEST was developed in 2013 by Dr. Homma, a co-author of this study, at Tokyo Medical University to teach trauma surgery techniques through a combination of lectures and cadaver-based practical training. Between October 2017 and December 2023, we conducted eleven 1-day seminars to teach trauma surgery techniques. We used 2–5 Thiel-embalmed cadavers for each course [17, 18], with one cadaver per 4–7 participants. Following the C-BEST program [15], 21 trauma procedures taught, included ① cricothyroidotomy; ② chest tube insertion; ③ fasciotomy of the lower extremity; ④ exposure of femoral vessels; ⑤ exposure of neck vessels; ⑥ vascular repair (direct suture, patch repair, end-to-end anastomosis, and shunting); ⑦ pericardial window technique; ⑧ left anterior thoracotomy and aortic clamping; ⑨ bilateral anterior thoracotomy (clamshell); ⑩ pulmonary hilar clamping; ⑪ pulmonary injury; ⑫ atrial injury; ⑬ ventricular injury; ⑭ trauma laparotomy; ⑮ portal triad clamping (Pringle manoeuvre); ⑯ liver packing; ⑰ left medial visceral rotation (Mattox manoeuvre); ⑱ right medial visceral rotation (Cattell–Braasch manoeuvre); ⑲ nephrectomy; ⑳ abdominal damage control technique; and  pelvic packing. The timeframe for this training is shown in Fig. 1, which includes the detailed schedule of the eleven 1-day seminars. Each session followed a structured format that integrated didactic and interactive components into the hands-on training as follows:

- Before each practical session, all participants gathered for a brief lecture by the lead instructor covering the procedure, anatomical details, and key technical points.
- Participants were then divided into small groups to perform surgical techniques under instructor guidance.
- Each session concluded with a group-based feedback discussion to reinforce learning.
- The day ended with a final debriefing session to address remaining questions and facilitate discussion.

Participants were recruited through the Hokkaido University website. Interested individuals were allowed to apply without restrictions based on specialty, postgraduate years, trauma surgery experience, or previous course attendance. Participants provided information on their specialty, postgraduate years, trauma surgery experience, training experience, and self-assessment of confidence levels (SACL) in performing the 21 training techniques. SACL assessments were conducted before, immediately after, and 6 months after the course.

C-BEST instructors certified by the C-BEST director as experienced and capable of teaching trauma surgery provided instruction. Surgeons with prior C-BEST experience who wished to assist joined as assistant instructors.

### Survey

Before participation, trainees reported their postgraduate year, specialty, total number of surgeries performed (selected from 10 categories: 0, 1–5, 6–10, 11–20, 21–50, 51–100, 101–200, 201–500, 501–1,000, and over 1,000), and experience with various surgical areas (head, neck, chest, abdomen, and limbs) across eight categories for each role (lead surgeon, first assistant with guidance, first assistant without guidance, or other assistant). Participants also rated their confidence in performing the 21 trauma procedures (SACL) on an 11-point Likert scale (0: not at all capable, 5: capable with an experienced assistant, 10: capable with a novice assistant). Surveys were completed before (pre), immediately after (post), and 6 months post-training (6 months later). Google Forms (Google LLC, Menlo Park, CA, USA) was used to collect responses, analysing data only from participants who provided answers at all three points.

### Statistical analyses

JMP® 17 (JMP, Cary, NC, USA) was used for statistical processing and evaluation. Background factors, including specialty and surgical volume, were compared between the novice (≤ 10 years post-graduation) and experienced (≥ 11 years post-graduation) groups using the chi-square or Fisher’s exact test when the expected frequencies were low. The SACL at each time point and changes in SACL over time were compared between the two groups using the Wilcoxon rank-sum test with significance set at *p* < 0.05. The impact of postgraduate year on total SACL at each time point and changes in total SACL over time were evaluated using univariate regression analysis.

## Results

### Overview

A total of 128 participants attended the 11 seminars and completed the survey. Of these, 117 participants (91.4%) completed all three surveys (pre-course, post-course, and 6 months later), and their data were analysed.

### Participant background

**Fig. 2:**
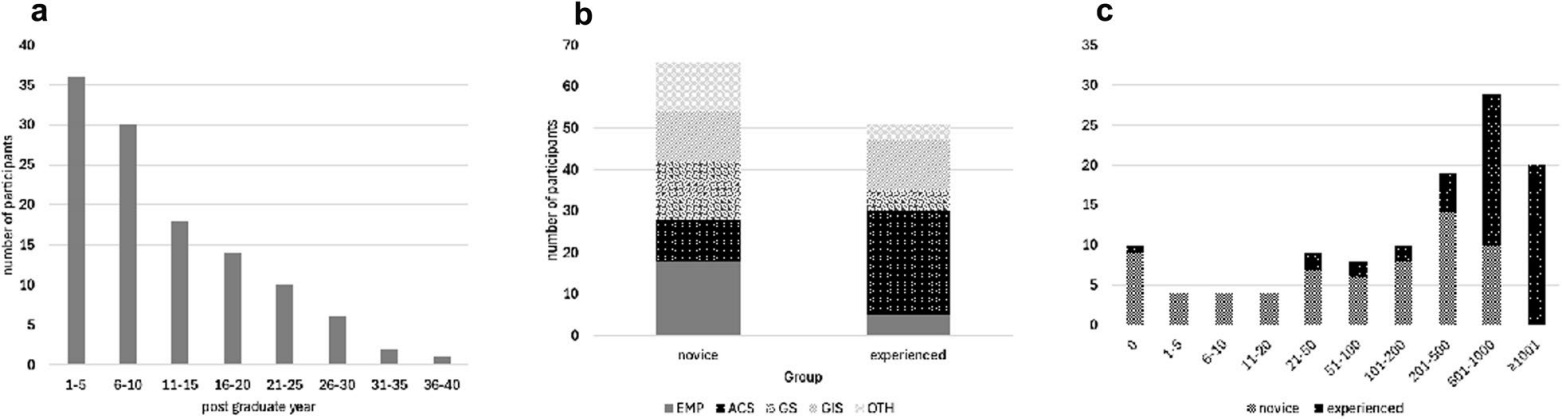
Participants’ backgrounds. **a** The median postgraduate year of the participants is 8 years, ranging from 2 to 36 years. For the novice group, the median is 5 years (range: 2–10 years), while it is 19 years (range: 11–36 years) for the senior group. **b** In terms of specialty, ACSs are the most common among all participants and within the senior group, whereas EMPs are the most prevalent among the novice group. A significant difference is observed in the distribution of specialties between the two groups. **c** The overall median range for the total number of surgeries performed is 201–500 cases. The median range is 51–100 cases for the novice group and 501–1,000 cases for the senior group, showing a significant difference in distribution between the two groups. *EMPs* emergency medicine physicians, *ACSs* acute care surgeons, *GSs* general surgeons, *GISs* gastrointestinal surgeons, *OTH* others

The median postgraduate years of the entire cohort was 8 years [range, 2–36 years] (5 years [2–10 years] for the novice group and 19 years [11–36 years] for the experienced group) (Fig. 2a). The most common specialty among all participants and experienced surgeons was Acute Care Surgery, whereas Emergency Medicine was the most common among novices, with a significant difference in distribution between the groups (Fig. 2b). The median range for the total surgeries performed was 201–500 cases; novices had 51–100 cases, while experienced surgeons had 501–1,000 cases, with a significant distribution difference (Fig. 2c).

### SACL analysis across groups

**Fig. 3:**
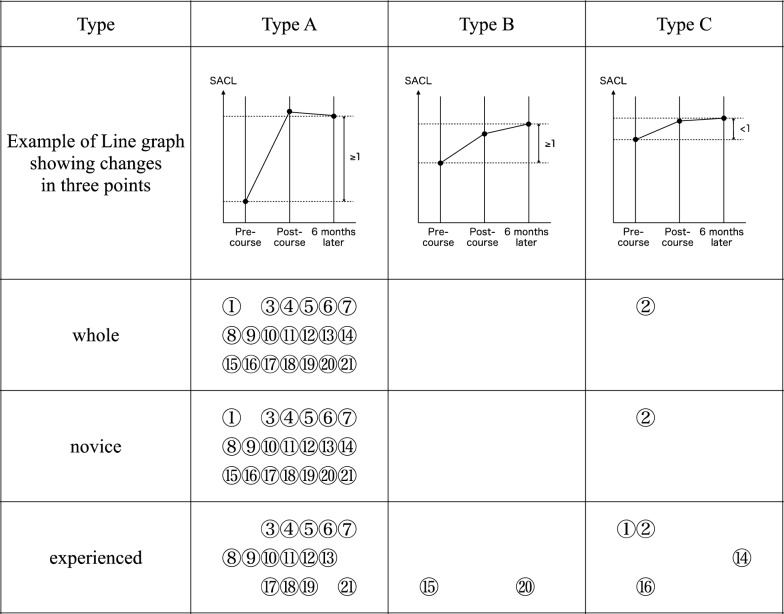
Classification of changes in SACL by type for each group. Classification of changes in SACL before, immediately after, and 6 months after the course is shown. In Type A, the average SACL increase is at least 1 point between pre- and post-course and between pre- and 6 months later. Type B also shows more than 1 point increase on average between pre- and 6 months later and an average increase of less than 1 point between pre- and post-course. In Type C, SACL increase is less than 1 point on average between pre- and post-course and between pre- and 6 months later

The median SACL for each item and time point was significantly higher in the experienced group than in the novice group, except for chest tube insertion post-course (Table 1). Changes in SACL across the time points showed three patterns: Type A (average increase of at least 1 point between pre- and post-course, and between pre-course and after 6 months), Type B (less than 1 point average increase between pre- and post-course, but more than 1 point average increase between pre-course and 6 months later), and Type C (no increase of more than 1 point on average between pre- and post-course, or between pre-course and 6 months later) (Table 1 and Fig. 3).

**Table 1:** SACL values and changes for each surgical technique

Surgical procedure		SACL median [Interquartile range]						SACL change average [Min, Max]]						Type of change in SACL
		Pre-course		Post-course		6-month later		Pre vs. Post		Pre vs. 6-month later		Post vs. 6-month later		
Basic technique														
①	Cricothyroidotomy	8.0 [5.0–10.0]		9.0 [8.0–10.0]		9.0 [8.0–10.0]		1.3 [−2.0, 7.0]		1.2 [−2.0, 6.0]		−0.1 [−5.0, 4.0]		A
		7.0 [5.0–9.0]	]^*^	8.0 [7.0–10.0]	]^*^	8.0 [6.3–10.0]	]*	1.9 [−2.0, 7.0]	]^*^	1.7 [−2.0, 6.0]	]^*^	−0.2 [−3.0, 4.0]		A
		10.0 [8.0–10.0]		10.0 [8.5–10.0]		10.0 [9.0–10.0]		0.5 [−2.0, 5.0]		0.5 [−2.0, 5.0]		0.0 [−5.0, 4.0]		C
②	Chest tube insertion	10.0 [8.0–10.0]		10.0 [9.0–10.0]		10.0 [9.0–10.0]		0.3 [−2.0, 4.0]		0.3 [−2.0, 5.0]		0.1 [−3.0, 3.0]		C
		10.0 [8.0–10.0]	]^*^	10.0 [9.0–10.0]		10.0 [8.3–10.0]	]*	0.5 [−2.0, 4.0]	]*	0.4 [−2.0, 5.0]		0.0 [−3.0, 3.0]		C
		10.0 [10.0–10.0]		10.0 [9.5–10.0]		10.0 [10.0–10.0]		0.0 [−2.0, 3.0]		0.2 [−2.0, 3.0]		0.2 [−2.0, 3.0]		C
Injuries to the extremities														
③	Fasciotomy of the lower extremity	5.0 [1.0–6.0]		7.0 [5.0–8.0]		6.0 [4.0–8.0]		2.1 [−4.0, 8.0]		1.6 [−3.0, 10.0]		−0.5 [−5.0, 4.0]		A
		3.0 [0.0–5.0]	]^*^	5.5 [4.0–7.8]	]^*^	5.0 [3.3–6.0]	]^*^	2.7 [−4.0, 8.0]	]^*^	1.9 [−3.0, 7.0]	]^*^	−0.8 [−5.0, 4.0]	]^*^	A
		5.0 [5.0–8.5]		8.0 [6.5–8.0]		8.0 [5.0–9.0]		1.3 [−3.0, 8.0]		1.2 [−2.0, 10.0]		−0.1 [−4.0, 3.0]		A
Vascular trauma														
④	Exposure of femoral vessels	5.0 [3.0–8.0]		7.0 [5.0–9.0]		7.0 [5.0–9.0]		1.5 [−8.0, 7.0]		1.4 [−2.0, 8.0]		−0.1 [−4.0, 6.0]		A
		4.0 [2.0–6.0]	]^*^	6.0 [5.0–7.0]	]^*^	5.0 [4.3–8.0]	]^*^	1.9 [−2.0, 6.0]	]^*^	1.6 [−2.0, 8.0]	]^*^	−0.3 [−4.0, 4.0]		A
		8.0 [5.0–10.0]		8.0 [7.0–10.0]		9.0 [6.5–10.0]		1.0 [−8.0, 7.0]		1.0 [−2.0, 7.0]		0.1 [−3.0, 6.0]		A
⑤	Exposure of neck vessels	5.0 [2.0–7.0]		7.0 [5.0–8.0]		6.0 [5.0–8.0]		2.0 [−4.0, 10.0]		1.7 [−3.0, 8.0]		−0.3 [−4.0, 5.0]		A
		3.0 [0.3–5.0]	]^*^	6.0 [5.0–7.0]	]^*^	5.0 [4.0–6.8]	]^*^	2.4 [−2.0, 8.0]	]^*^	1.9 [−3.0, 6.0]		−0.5 [−4.0, 3.0]		A
		6.0 [4.0–10.0]		8.0 [7.0–10.0]		8.0 [6.0–10.0]		1.5 [−4.0, 10.0]		1.4 [−3.0, 8.0]		−0.1 [−3.0, 5.0]		A
⑥	Vascular repair (direct suture, patch repair, end-to-end anastomosis, shunting)	3.0 [1.0–6.0]		6.0 [4.0–7.0]		5.0 [3.0–7.0]		2.0 [−2.0, 8.0]		1.8 [−2.0, 9.0]		−0.2 [−5.0, 6.0]		A
		1.0 [0.0–3.0]	]^*^	5.0 [3.0–6.0]	]^*^	4.5 [3.0–6.0]	]^*^	2.4 [−1.0, 7.0]	]^*^	2.2 [−1.0, 9.0]	]^*^	−0.2 [−5.0, 6.0]		A
		5.0 [3.0–9.0]		7.0 [6.0–9.0]		7.0 [5.0–8.0]		1.4 [−2.0, 8.0]		1.2 [−2.0, 8.0]		−0.2 [−3.0, 3.0]		A
Thoracic trauma														
⑦	Pericardial window technique	5.0 [2.0–8.0]		7.0 [5.0–9.0]		7.0 [5.0–9.0]		1.8 [−3.0, 8.0]		1.7 [−3.0, 8.0]		−0.1 [−6.0, 4.0]		A
		4.0 [0.3–5.0]	]^*^	6.0 [5.0–8.0]	]*	5.5 [4.3–8.0]	]*	2.3 [−2.0, 8.0]	]*	2.2 [−2.0, 7.0]	]*	−0.1 [−4.0, 4.0]		A
		8.0 [5.0–10.0]		8.0 [6.0–10.0]		8.0 [6.0–10.0]		1.2 [−3.0, 8.0]		1.1 [−3.0, 8.0]		−0.1 [−6.0, 3.0]		A
⑧	Left anterior thoracotomy & aortic clamping	6.0 [2.0–10.0]		8.0 [6.0–9.0]		8.0 [5.0–10.0]		1.4 [−3.0, 8.0]		1.5 [−5.0, 8.0]		0.0 [−3.0, 5.0]		A
		5.0 [1.3–8.0]	]^*^	7.0 [5.0–8.0]	]*	6.5 [5.0–8.0]	]*	1.6 [−2.0, 7.0]		1.6 [−5.0, 7.0]		0.0 [−3.0, 4.0]		A
		8.0 [5.0–10.0]		8.0 [7.0–10.0]		9.0 [7.5–10.0]		1.2 [−3.0, 8.0]		1.3 [−3.0, 8.0]		0.1 [−2.0, 5.0]		A
⑨	Bilateral anterior thoracotomy (clam shell)	5.0 [1.0–8.0]		7.0 [5.0–9.0]		7.0 [5.0–9.0]		1.9 [−3.0, 8.0]		1.7 [−2.0, 8.0]		−0.3 [−5.0, 5.0]		A
		4.0 [0.0–6.0]	]^*^	6.0 [5.0–8.0]	]*	5.0 [4.0–8.0]	]*	2.3 [−2.0, 8.0]	]*	1.9 [−2.0, 8.0]	]*	−0.4 [−5.0, 3.0]		A
		8.0 [3.5–10.0]		8.0 [6.5–10.0]		9.0 [6.5–10.0]		1.4 [−3.0, 8.0]		1.4 [−2.0, 8.0]		−0.1 [−4.0, 5.0]		A
⑩	Pulmonary hilar clamping	5.0 [1.0–8.0]		7.0 [5.0–8.0]		6.0 [4.0–8.0]		1.9 [−3.0, 10.0]		1.7 [−3.0, 8.0]		−0.2 [−5.0, 4.0]		A
		2.0 [0.0–5.0]	]^*^	5.5 [3.3–7.0]	]*	5.0 [3.0–7.8]	]*	2.3 [−2.0, 10.0]	]*	2.0 [−3.0, 8.0]	]*	−0.3 [−5.0, 4.0]		A
		7.0 [3.0–10.0]		8.0 [6.5–10.0]		8.0 [6.0–10.0]		1.3 [−3.0, 8.0]		1.4 [−2.0, 8.0]		0.0 [−3.0, 3.0]		A
⑪	Pulmonary injury	5.0 [1.0–7.0]		7.0 [5.0–8.0]		6.0 [4.0–8.0]		2.2 [−4.0, 10.0]		1.8 [−2.0, 8.0]		−0.4 [−6.0, 5.0]		A
		2.0 [0.0–5.0]	]^*^	6.0 [4.3–8.0]	]*	5.0 [3.0–7.0]	]*	2.8 [−4.0, 6.0]	]*	2.1 [−2.0, 6.0]	]*	−0.6 [−6.0, 4.0]		A
		6.0 [4.0–9.5]		8.0 [6.0–10.0]		8.0 [5.0–10.0]		1.5 [−2.0, 10.0]		1.3 [−2.0, 8.0]		−0.2 [−3.0, 5.0]		A
⑫	Atrial injury	3.0 [0.0–5.0]		6.0 [3.0–7.0]		5.0 [3.0–7.0]		2.0 [−3.0, 8.0]		1.5 [−3.0, 8.0]		−0.5 [−5.0, 4.0]		A
		1.0 [0.0–4.0]	]^*^	5.0 [3.0–6.0]	]*	4.0 [2.0–5.0]	]*	2.3 [−3.0, 6.0]	]*	1.7 [−2.0, 6.0]		−0.6 [−5.0, 4.0]		A
		5.0 [2.0–8.5]		7.0 [5.5–8.0]		7.0 [5.0–8.0]		1.6 [−2.0, 8.0]		1.2 [−3.0, 8.0]		−0.4 [−4.0, 4.0]		A
⑬	Ventricular injury	3.0 [0.0–5.0]		6.0 [3.0–7.0]		5.0 [3.0–7.0]		2.1 [−3.0, 8.0]		1.6 [−4.0, 8.0]		−0.4 [−5.0, 4.0]		A
		1.5 [0.0–4.0]	]*	5.0 [3.0–6.0]	]*	4.0 [2.0–5.0]	]*	2.3 [−3.0, 6.0]	]*	1.8 [−3.0, 7.0]		−0.5 [−5.0, 4.0]		A
		5.0 [2.0–8.0]		7.0 [5.5–8.0]		7.0 [5.0–8.0]		1.8 [−2.0, 8.0]		1.4 [−4.0, 8.0]		−0.3 [−3.0, 4.0]		A
Abdominal & pelvic trauma														
⑭	Trauma laparotomy	6.0 [3.0–10.0]		8.0 [6.0–10.0]		8.0 [5.0–10.0]		1.5 [−3.0, 9.0]		1.3 [−4.0, 9.0]		−0.3 [−6.0, 4.0]		A
		5.0 [1.3–6.8]	]^*^	7.0 [5.0–8.8]	]^*^	6.0 [4.0–8.0]	]^*^	2.3 [−2.0, 9.0]	]^*^	1.8 [−4.0, 9.0]	]^*^	−0.6 [−6.0, 3.0]	]^*^	A
		9.0 [7.0–10.0]		10.0 [8.0–10.0]		10.0 [8.0–10.0]		0.5 [−3.0, 5.0]		0.7 [−1.0, 5.0]		0.2 [−3.0, 4.0]		C
⑮	Portal triad clamping (Pringle’s maneuver)	5.0 [2.0–8.0]		8.0 [5.0–10.0]		7.0 [5.0–10.0]		1.7 [−4.0, 8.0]		1.5 [−5.0, 6.0]		−0.2 [−5.0, 3.0]		A
		4.5 [1.0–6.0]	]^*^	7.0 [5.0–8.0]	]^*^	5.0 [4.0–8.0]	]^*^	2.3 [−4.0, 8.0]	]^*^	1.9 [−5.0, 6.0]	]^*^	−0.4 [−5.0, 3.0]	]^*^	A
		8.0 [6.0–10.0]		10.0 [8.0–10.0]		10.0 [8.0–10.0]		0.8 [−2.0, 5.0]		1.0 [−1.0, 5.0]		0.1 [−2.0, 2.0]		B
⑯	Liver packing	6.0 [3.0–8.0]		8.0 [6.0–9.0]		8.0 [5.0–10.0]		1.6 [−2.0, 9.0]		1.7 [−3.0, 8.0]		0.1 [−3.0, 3.0]		A
		5.0 [1.0–6.0]	]^*^	7.0 [5.0–8.0]	]^*^	7.0 [5.0–8.0]	]^*^	2.4 [−2.0, 9.0]	]^*^	2.4 [−3.0, 8.0]	]^*^	0.0 [−3.0, 3.0]		A
		8.0 [7.0–10.0]		8.0 [7.5–10.0]		9.0 [8.0–10.0]		0.6 [−2.0, 5.0]		0.8 [−2.0, 5.0]		0.2 [−2.0, 2.0]		C
⑰	Left medial visceral rotation (Mattox maneuver)	4.0 [0.0–7.0]		7.0 [4.0–8.0]		6.0 [4.0–9.0]		1.9 [−2.0, 10.0]		1.9 [−1.0, 10.0]		0.0 [−4.0, 5.0]		A
		2.0 [0.0–5.0]	]^*^	5.0 [3.0–7.0]	]^*^	5.0 [3.0–7.0]	]^*^	2.4 [−2.0, 10.0]	]^*^	2.3 [−1.0, 10.0]	]^*^	−0.1 [−4.0, 5.0]		A
		7.0 [4.5–10.0]		8.0 [6.5–10.0]		8.0 [7.0–10.0]		1.3 [−2.0, 10.0]		1.4 [−1.0, 10.0]		0.1 [−2.0, 2.0]		A
⑱	Right medial visceral rotation (Cattell–Braasch maneuver)	4.0 [0.0–7.0]		7.0 [4.0–8.0]		6.0 [4.0–9.0]		2.0 [−2.0, 10.0]		1.9 [−1.0, 10.0]		0.0 [−4.0, 5.0]		A
		2.0 [0.0–5.0]	]^*^	5.0 [3.0–7.0]	]^*^	5.0 [3.0–6.8]	]^*^	2.5 [−2.0, 10.0]	]^*^	2.4 [−1.0, 10.0]	]^*^	−0.1 [−4.0, 5.0]		A
		7.0 [4.5–10.0]		8.0 [6.5–10.0]		8.0 [7.0–10.0]		1.3 [−2.0, 10.0]		1.4 [−1.0, 10.0]		0.1 [−2.0, 2.0]		A
⑲	Nephrectomy	3.0 [0.0–7.0]		6.0 [3.0–8.0]		5.0 [3.0–8.0]		1.7 [−3.0, 10.0]		1.7 [−3.0, 10.0]		0.0 [−5.0, 9.0]		A
		1.0 [0.0–4.0]	]^*^	5.0 [2.3–6.0]	]^*^	4.0 [3.0–6.0]	]^*^	2.2 [−2.0, 10.0]	]^*^	2.2 [−2.0, 10.0]	]^*^	0.0 [−4.0, 9.0]		A
		7.0 [3.0–9.5]		8.0 [5.0–10.0]		8.0 [5.5–9.5]		1.2 [−3.0, 10.0]		1.1 [−3.0, 10.0]		−0.1 [−5.0, 2.0]		A
⑳	Abdominal damage control technique	5.0 [2.0–8.0]		7.0 [5.0–9.0]		7.0 [5.0–9.0]		1.7 [−4.0, 10.0]		1.6 [−3.0, 10.0]		−0.1 [−5.0, 5.0]		A
		4.0 [1.0–5.8]	]^*^	6.0 [4.0–8.0]	]^*^	5.0 [4.0–7.8]	]^*^	2.3 [−2.0, 9.0]	]^*^	2.0 [−3.0, 7.0]	]^*^	−0.3 [−5.0, 5.0]		A
		8.0 [5.0–9.5]		8.0 [7.0–10.0]		9.0 [7.0–10.0]		0.9 [−4.0, 10.0]		1.1 [−3.0, 10.0]		0.2 [−2.0, 3.0]		B
	Pelvic packing	5.0 [2.0–7.0]		7.0 [5.0–9.0]		7.0 [5.0–9.0]		2.1 [−6.0, 10.0]		2.0 [−5.0, 10.0]		−0.1 [−5.0, 6.0]		A
		3.0 [1.0–5.0]	]^*^	6.0 [5.0–8.0]	]^*^	5.0 [4.0–7.8]	]^*^	2.7 [−6.0, 9.0]	]^*^	2.4 [−5.0, 8.0]	]^*^	−0.3 [−5.0, 3.0]		A
		7.0 [5.0–10.0]		8.0 [7.0–10.0]		9.0 [7.0–10.0]		1.5 [−5.0, 10.0]		1.6 [−3.0, 10.0]		0.1 [−5.0, 6.0]		A
Sum of all items														
		100.0 [54.0–144.0]		149.0 [115.0–172.0]		136.0 [104.0–173.0]		36.8 [−46.0, 144.0]		33.2 [−23.0, 142.0]		−3.6 [−67.0, 59.0]		
		74.0 [30.5–113.0]	]^*^	134.0 [92.3–151.8]	]^*^	114.5 [85.8–148.5]	]^*^	46.8 [−46.0, 132.0]	]^*^	40.5 [−14.0, 106.0]	]^*^	−6.3 [−67.0, 48.0]		
		138.0 [94.0–199.5]		167.0 [147.5–194.0]		172.0 [136.5–198.5]		23.8 [−42.0, 144.0]		23.6 [−23.0, 142.0]		−0.2 [−39.0, 59.0]		

On the left side of the table, the median SACL values [interquartile range] for each surgical skill at three points in time (pre-course, post-course, and 6 months later) are shown. On the right side, the average [minimum–maximum] changes in SACL from pre-course to post-course, pre-course to 6 months later, and post-course to 6 months later are shown for each training item and the total of all training items. The upper row for each item shows the overall results, the middle row shows the results for the novice group (≤ 10 years post-graduation), and the lower row shows the results for the experienced group (≥ 11 years post-graduation). Significant differences in the comparison between the novice and experienced groups are indicated by an asterisk (* *p* < 0.05)

SACL increases from pre- to post-course were significantly greater in the novice group than in the experienced group for items ①–⑦, ⑨–, and total SACL. SACL increases from pre-course to 6 months later were also significantly greater in the novice group for items ①, ③, ④, ⑥, ⑦, ⑨-⑪, ⑭-, and total SACL. Comparisons between post-course and 6 months later SACL showed significant declines in the novice group for items ③, ⑭, and  (Table 1).

**Fig. 4:**
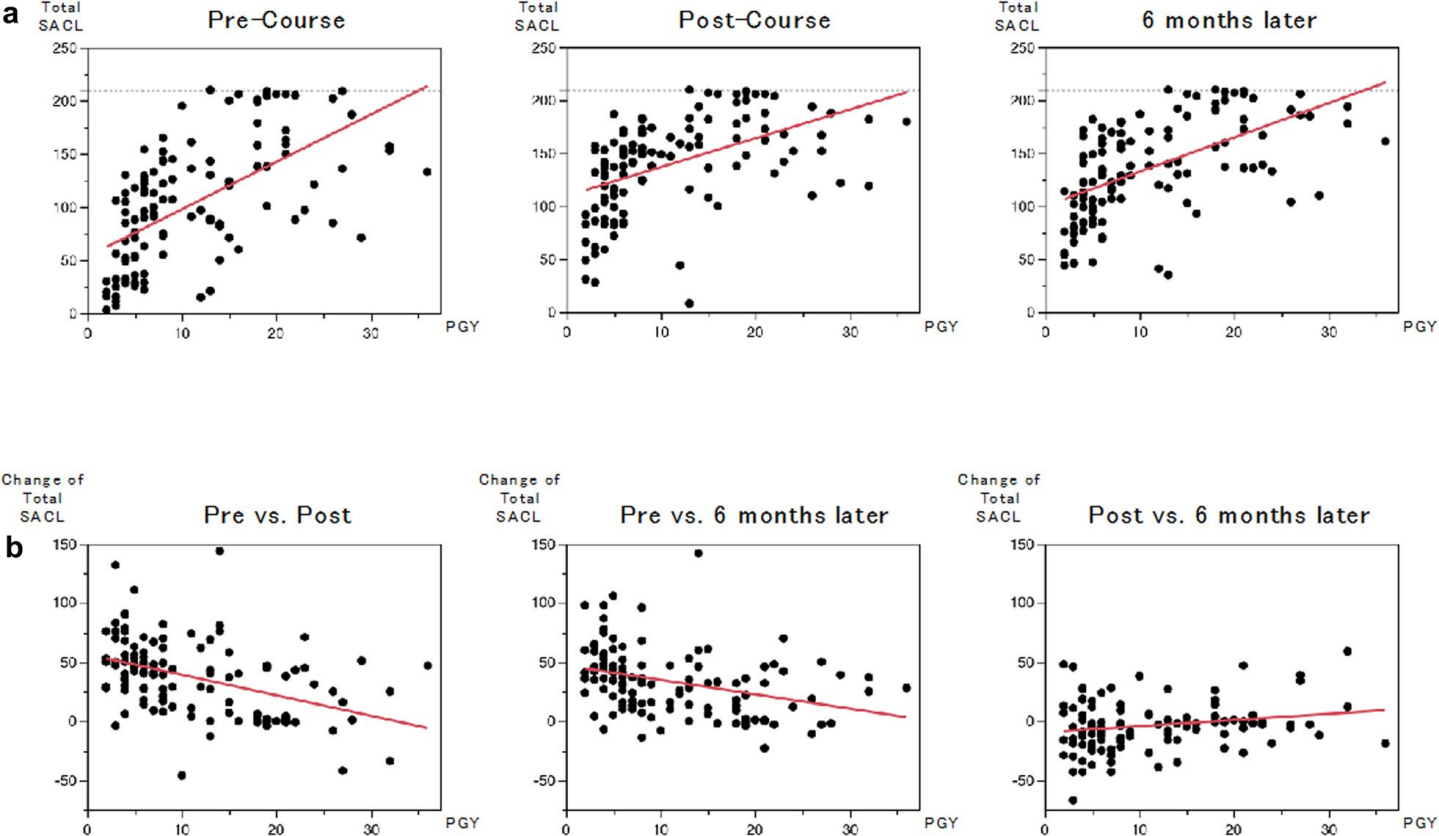
Regression analysis between postgraduate years and total SACL scores. **a** Regression analysis between postgraduate years and total SACL scores revealing a positive correlation at all time points: pre-course (slope: 4.45, 95% CI: 3.36–5.53, R^2^ = 0.37), post-course (slope: 2.71, 95% CI: 1.83–3.59, R^2^ = 0.24), and 6 months later (slope: 3.23, 95% CI: 2.37–4.10, R^2^ = 0.32).** b** Regression analysis of changes in the total SACL scores with respect to postgraduate years showing a weak negative correlation between pre-course and post-course (slope: − 1.73, 95% CI: − 2.39 to − 1.07, R^2^ = 0.19) and between pre-course and 6 months later (slope: − 1.21, 95% CI: − 1.81 to − 0.62, R^2^ = 0.12). A weak positive correlation is observed between post-course and 6 months later (slope: 0.52, 95% CI: 0.05–0.99, R.^2^ = 0.04)

Correlation analysis between postgraduate years and total SACL showed positive correlations at all time points (pre-course: r = 0.61, post-course: r = 0.50, 6 months later: r = 0.57) (Fig. 4a). Analysis of changes in total SACL revealed weak negative correlations between pre- and post-course (r =  − 0.44) and between pre-course and 6 months (r =  − 0.35), with no significant correlation between post-course and 6 months later (r = 0.20) (Fig. 4b).

## Discussion

In this study, we evaluated the effectiveness of trauma surgical skill training using the C-BEST program for a wide range of clinicians, from novices to experienced surgeons. The results showed a significant improvement in participants’ confidence levels (SACL) immediately after the course, with the effects maintained 6 months later. Additionally, a positive correlation was observed between postgraduate years and total SACL at all time points, indicating that the longer the years of surgical experience, the higher the confidence.

Unlike animal-based courses, such as The Advanced Trauma Operative Management (ATOM) Course [19, 20], in which the necessity of maintaining animal viability restricts the depth of instruction and feedback, cadaver-based training allows for extended feedback and discussion, potentially enhancing learning outcomes. This ability to integrate structured instruction and immediate feedback may explain the sustained improvement in confidence observed among participants.

The changes in SACL could be classified into three types. Almost all items showed an average increase of > 1 in SACL immediately after the course and 6 months later compared to before the course, and these were classified as Type A. The SACL increase in Type A indicated that the training items in C-BEST were appropriate. Type B showed no significant increase in SACL immediately after the course, but after 6 months, an average increase of more than 1 was observed, and this was due to two items of the course (“portal triad clamping” and “abdominal damage control technique”) in the experienced group. As the experienced surgeons had the opportunity to perform similar procedures as in their regular practice, their SACL increased over time, which is also believed to show the effectiveness of attending C-BEST. Cricothyroidotomy, chest tube insertion, trauma laparotomy, and liver packing procedures are relatively simple; however, because the surgeons’ initial SACL was high, it created a ceiling effect. Therefore, it was determined that these items could be removed from the training items in C-BEST for relevant groups.

The correlation analysis between postgraduate years and changes in SACL showed that younger clinicians experienced significant improvement immediately after the course, whereas experienced surgeons maintained relatively stable confidence. This indicates that C-BEST provides an opportunity for novice clinicians to rapidly acquire new skills and build confidence, while for experienced surgeons, it serves as a place to reinforce and reaffirm existing skills.

Many emergency physicians who do not routinely perform surgical procedures have participated in C-BEST. They previously encountered preventable trauma deaths due to the lack of surgical support and attended C-BEST to better prepare for such situations. While some techniques, particularly those involving suturing, require extensive instruction, most trauma procedures rely on fundamental dissection and separation skills, making them feasible for emergency physicians to acquire. Some emergency physicians have successfully applied C-BEST-acquired techniques to save critically injured patients, while others pursued further surgical training and transitioned into Acute Care Surgeons. These findings highlight the importance of C-BEST in preparing emergency physicians for critical trauma situations, particularly in environments with limited surgical support.

This study had some limitations. First, the sample size was limited, and the analysis was restricted to data from Hokkaido University; thus, caution is needed when generalizing the results to other facilities or regions that offer the C-BEST program. Additionally, since SACL is based on self-assessment, using other objective measures (e.g., Objective Structured Assessment of Technical Skills (OSATS) scores or surgical performance evaluations) alongside would better evaluate skill improvement. Furthermore, the 6-month follow-up after the training is relatively short; long-term follow-up is needed. Future studies should verify the long-term effects of the C-BEST program using objective indicators, such as surgical outcomes in clinical practice and patient outcomes.

To enhance C-BEST’s effectiveness, future research could explore methods to integrate C-BEST training into clinical simulation environments, such as virtual reality or augmented reality platforms. These technologies could provide participants with more frequent and accessible practice opportunities, particularly for techniques that showed a Type A pattern, where confidence waned after 6 months. Additionally, implementing periodic refresher courses or on-demand online modules can address skill decay and help maintain long-term confidence.

Another area for improvement is the evaluation of the impact of C-BEST on actual clinical outcomes. Future studies could collect data on patient outcomes and surgical performance metrics from C-BEST participants in clinical settings to quantify the real-world benefits of the program. This approach would provide a more comprehensive assessment of the utility of C-BEST beyond self-reported confidence levels.

Finally, expanding C-BEST to include interdisciplinary training with emergency medical technicians or paramedics could enhance teamwork skills that are essential in trauma care. Investigating how multidisciplinary C-BEST sessions affect the confidence and skills of both surgical and non-surgical professionals would be a valuable addition to the current research.

## Conclusions

In summary, the results of this study confirm that C-BEST is effective as a means of lifelong education, providing a critical means for young clinicians to acquire skills and contributing to skill maintenance and enhancement for experienced surgeons. Improving the program by following up participants and collecting feedback is necessary.

## Data Availability

The data that support the findings of this study are available on request from the corresponding author.

## Abbreviations

EMPs: Emergency medicine physicians
ACSs: Acute care surgeons
GSs: General surgeons
GISs: Gastrointestinal surgeons
OSATS: Objective structured assessment of technical skills
OTH: Others
C-BEST: Cadaver-based educational seminar for trauma
SACL: Surgery self-assessment of confidence levels scale
